# Commentary: Coronavirus and Obesity: Could Insulin Resistance Mediate the Severity of Covid-19 Infection?

**DOI:** 10.3389/fpubh.2020.00351

**Published:** 2020-07-07

**Authors:** Vincenza Frisardi

**Affiliations:** Geriatric and NeuroRehabilitation Department, Clinical and Nutritional Lab, AUSL-IRCCS Arcispedale Santa Maria Nuova, Reggio Emilia, Italy

**Keywords:** COVID-19, insulin resistance, metabolic syndrome (MES), obesity, non-alchoholic fatty liver disease

The emerging SARS-Coronavirus 2 (SARS-CoV-2) is a worldwide public health emergency. Understanding the molecular mechanisms related to the higher risk both of being infected by SARS-CoV-2 and of developing a more serious disease (COVID-19) could be useful for developing therapeutic interventions.

## Introduction

Insulin Resistance (IR) might be a potential key factor behind the COVID-19 severity found in people with obesity. An article published on Frontiers Public Health supported the evidence of possible mechanisms linking IR and COVID-19 severity via the upregulation of ACE 2, the protein involved in virus entry (1). This research area is worthy of being investigated further for its implication in the prevention and treatment of this dramatic pandemic. We need to understand the molecular mechanisms related to the higher risk both of being infected by SARS-CoV-2 and of developing a more severe disease (COVID-19).

## Preliminary Report on Metabolic Disorders and COVID-19

Recent reports of hospitalized COVID-19 patients have found obesity to be a risk factor for the worst adverse outcomes (severity and mortality). The Intensive Care National Audit and Research Centre (ICNARC) report on 2,621 patients in intensive care units in England showed that the case fatality rate was higher in obese patients^1^. The International Severe Acute Respiratory & Emerging Infection Consortium (ISARIC) International report of 1,123 patients with suspected or confirmed COVID-19 found that obesity was the fifth most observed comorbidity in hospitalized patients—only somewhat less common than “high-risk” pulmonary conditions^2^. Systematic reviews and meta-analyses confirmed, surprisingly, that metabolic disorders seem to play a more pivotal role for negative outcomes in COVID-19 compared with preexistent chronic respiratory disease (2). Starting from this, it would be appropriate to think of obesity in relation to COVID-19 outcome in a more complex way, rather than considering only the mechanical effects of abdominal compression on the respiratory dynamic.

## Insulin and COVID-19: Suggestion for Underlying Mechanisms

Finucane and Davenport argued that the insulin-mediated metabolic and inflammatory processes could be the cause of the negative SARS-CoV2-related trajectory in obese patients. In the last few decades, insulin was believed to intervene in other degenerative diseases both as a principal leading factor and in a cross-talk with other metabolic disorders (3, 4). Because obesity and IR have a bidirectional relationship and the adipose-insulin axis was postulated (5, 6), the research needs to be more addressed toward the convoluted route linking lipid and glucose metabolism as a unique molecular platoon. Insulin is a critical regulator of many cellular pathways, with many already demonstrated tissue-specific actions. Rapid changes in protein phosphorylation and function as well as changes in gene expression mediate the insulin-related metabolic effects (6). Finucane and Davenport reported the evidence that insulin-mediated ACE2 expression varies in a tissue-specific manner with significant expression in the lungs. Whether the high glucose level rather than elevated insulin levels is responsible for this overexpression is worthy of investigation because it might have clinical relevance. As reported from the authors, in people with obesity and diabetes, it is clear that other mechanisms independent of ACE2 expression are likely to contribute to the more severe phenotype of COVID-19.

The clinical manifestations of COVID-19 are heterogeneous, with the lungs the most triggered organ. Nevertheless, other clinical expressions of SARS-CoV2 were reported, suggesting an interesting hypothesis about the host-pathogen interaction via the metabolism^1^. TMPRSS2, the most accused protein involved in virus activation, has also been detected in other tissues playing a metabolic role, in particular in bile ducts and the pancreas (7). Furthermore, adipose tissue is not only a simple fat store tissue, but is also a somewhat active endocrine organ. Gender and age differences in peptides and hormones secreted were also reported (5). Likely, this might explain why older people and males are more at risk of developing a negative outcome.

## Discussion

Lipids are structural elements of viral and cellular membranes. Viruses induce the formation of novel cytoplasmic membrane structures and compartments, in which viral genome replication and assembly occurs with, in some cases, shielding from host innate immune response. For instance, several enveloped and non-enveloped viruses are cholesterol-dependent for entry into cells and their replication (8). Moreover, the sterol pathway is involved in other cases of virus infection (9). Viruses require not only membranes on which to replicate but also specific lipids; lipotoxicity in obesity might answer to these requirements. IR is the molecular feature of Metabolic Syndrome (MES), a cluster of metabolic risk factors for cardiovascular disease As an analogy, the global risk would not depend on the sum of every single factor, but is likely to be affected by the exponential and multiplicative elements (3). Therefore, to build an integrated pathogenetic model to be as extensive as possible is advisable. IR was found in patients with hepatitis C virus (HCV) infection and often leads to the development of type II diabetes (10). As role of MES in COVID-19 is not clear, patients need an accurate metabolic assessment. Finucane and Davenport concluded with suggestions for clinical implications for studying insulin action in relation to COVID-19 severity. Unfortunately, in the initial studies of COVID-19, no data about insulin determination, BMI, or other systematic metabolic determinations are available.

Currently, regarding the application in routine clinical practice, concerns arise about the feasibility of measuring IR in acutely ill patients. Furthermore, it is arguable how valid the measure could be in people who fell sick and then fasted for several days before admission to the hospital. Therefore, a non-invasive way to assess the long-term consequences of insulin and lipid impairment could be done through the screening of non-alcoholic fatty liver disease (NAFLD, the hepatic manifestation of MES and IR), i.e., through the use of Fibroscan (11). These preliminary observations are highlighting the need to intensively investigate IR and other components of MES in COVID-19 pathogenesis. For this purpose, advanced digital solutions (big data, artificial intelligence, machine learning) for the development of sophisticated real-world based algorithms must be promoted.

## Author Contributions

The author confirms being the sole contributor of this work and has approved it for publication.

## Conflict of Interest

The author declares that the research was conducted in the absence of any commercial or financial relationships that could be construed as a potential conflict of interest.

## Footnotes

^1^https://www.icnarc.org/About/Latest-News/2020/03/27/Report-On-775-Patients-Critically-Ill-With-COVID-19

^2^https://media.tghn.org/medialibrary/2020/04/ISARIC_Data_Platform_COVID-19_Report_8APR20.pdf
